# Breast self-examination practice and associated factors among female healthcare workers in Ethiopia: A systematic review and meta-analysis

**DOI:** 10.1371/journal.pone.0241961

**Published:** 2020-11-10

**Authors:** Birye Dessalegn Mekonnen

**Affiliations:** Department of Nursing, Teda health Science College, Gondar, Ethiopia; Guttmacher Institute, UNITED STATES

## Abstract

**Background:**

Breast cancer is common global public health problem. It is the principal cause of cancer related death. In Ethiopia, study findings regarding prevalence and associated factors of BSE among female healthcare workers have been inconsistently reported and highly variable. Thus, this systematic review and meta-analysis aimed to determine the pooled prevalence of breast self-examination practice and determinants in Ethiopia.

**Methods:**

A systematic search of PubMed, Medline, EMBASE, Global Health, Google Scholar, CINAHL and Scopus from April 2, 2020 to April 24, 2020. Data were extracted from articles included in the review using a data extraction tool which was adapted from the Joanna Briggs Institute. the quality of each included article was evaluated using the Newcastle-Ottawa scale. Data analysis was done using STATA 11. The Cochrane Q and I^2^ test were used to assess heterogeneity between the studies; and publication bias was assessed using funnel plots and Egger’s test. A random effects meta-analysis was computed to determine the pooled prevalence of breast self-examination. The determinants for breast self-examination were examined. Forest plots were used to present the prevalence and odds ratio with 95% confidence interval.

**Results:**

After reviewing 9605 studies, 12 studies involving 4129 female healthcare workers were included for this review and meta-analysis. The pooled prevalence of breast self-examination practice among healthcare workers in Ethiopia was 56.31% (95% CI: 44.37, 68.25). The subgroup analysis further revealed that the higher breast self-examination practice was observed among other healthcare workers, 58.60% (95% CI: 43.31, 73.90). Good knowledge (AOR = 3.02; 95% CI: 1.24, 7.35), positive attitude (AOR = 2.73; 95% CI: 1.95, 4.13) and family history of breast cancer (AOR = 3.21; 95% CI: 1.22, 6.52) were significantly associated with breast self-examination practice among healthcare workers.

**Conclusion:**

This meta-analysis found that nearly half of the female healthcare workers were not performed breast self-examination. The finding of this study suggests the need of strengthening early diagnosis of breast cancer and control strategies with a collaborative effort of policymakers and other concerned stakeholders.

## Introduction

Breast cancer (BC) is the most frequent cancer among women, and the principal cause of cancer related death [1, 2]; which is ranked the second leading cause of death from cancer in women [3, 4]. The incidence of breast cancer has been rising in both developed and developing world [4–6].

Breast cancer is a global concern affecting over 2 million women worldwide [2]. Globally, 2.4 million new breast cancer cases and 53,000 deaths were reported in 2015; Of which, 13% occurred in Africa [7]. According to World Health Organization (WHO), almost 58% of breast cancer related deaths occur in less developed countries [8]. In addition, breast cancer is responsible for 15 million disability adjusted life years (DALYs) worldwide [2].

Evidences from cancer registries revealed that breast cancer incidence in sub-Saharan Africa is on the increase [9–11]. A review literature indicated that Africa had the highest breast cancer mortality rate, with the highest incidence rates being recorded within the sub-Saharan African (SSA) [12]. Adoption of westernized lifestyle and behaviours resulting in later age of reproduction, changes in diet and reduction in physical activity among the African population was the factor that had been implicated in the increasing incidence of breast cancer in Africa [10, 13–15]. In Africa, addressing several challenges in the response to a growing cancer burden remains difficult due to poorly representative data on cancer in many settings [9, 16].

Though breast cancer is underreported as most women seek treatment from traditional healers before getting support from health facility, around 10,000 women in Ethiopia are estimated having breast cancer [17]. Recent evidence also indicated that the burden of breast cancer in Ethiopia is high with incidence rate of 32.9% of all women cancers diagnosed [18].

Though breast self-examination (BSE) is currently not recommended in high income countries where mammography services are available [19], early identification of breast cancer through BSE plays an important role to improve breast cancer outcome and survival in low income countries [20–22]. In resource limited settings and weak health systems, women are diagnosed in late stages. Thus, early detection based on awareness of early signs and symptoms should prioritize [8]. In Africa, breast cancer prevention and control is comparatively limited [5, 23]. In addition, registries which play a critical role in cancer surveillance are limited with only 43.4% countries have active cancer registries in SSA [24].

Breast self-examination practice is cost effective, convenient, painless, easy to apply, private, safe, and noninvasive screening methods made by each woman for early detection of breast cancer [25, 26]. The possibility of early and easily recognizing of any changes on the breast could achieved when women perform BSE on a regular basis [27, 28]; as most of the early breast tumors are self-discovered in SSA [29].

Though breast self-screening is recommended to detect abnormalities in developing countries, most of women do not perform BSE [30–33]. The identified factors that prevent breast self-screening including the absence of signs and symptoms, fear, lack of healthcare workers (HCW) recommendations, forgetting the schedule of BSE, pain, embarrassment, lack of conducive environment and cultural support, and the absence of support from spouse [34–41].

When healthcare workers counsel individuals with positive health behaviors, they must be role models to motivate their clients. Female healthcare workers therefore can be the best examples in educating and implementing their activities including breast self-screening. In addition, female healthcare workers are change agents who often offer useful counseling on health promotion especially for women, so that they could serve as role models.

In this study, literature on breast self-screening practice among female healthcare workers in Ethiopia were reviewed. However, the studies show a difference in practice and associated factors, and to the author knowledge, the literatures have not been examined systematically. Therefore, this systematic review and meta-analysis was aimed to estimate the pooled prevalence of breast self-examination practice and to identify associated factors among female healthcare workers in Ethiopia. The findings of this meta-analysis will help for policy makers, stakeholders and other concerned bodies to identify gaps in breast self-screening practice, and to plan strategies to increase the practice of breast self-screening. Moreover, it will help to plan and fight against unfavorable consequences of breast cancer.

## Methods

This systematic review and meta-analysis was carried out according to Preferred Reporting Items for Systematic Reviews and Meta-Analyses (PRISMA) checklist [42] (S1 Table).

### Search strategy and information sources

A systematic search of published literature was conducted through electronic databases including PubMed, Medline, EMBASE, Global Health, Google Scholar, Scopus, web of science and African journal online (AJOL) from April 2, 2020 to April 24, 2020. The search was done using keyword based on Medical Subject Headings (MeSH) with the following search terms: “breast self-examination” AND “practice” OR “exercise” AND “associated factors” OR “determinants” OR “predictors” AND “female health professionals” OR “female healthcare workers” AND “Ethiopia”. The search focused on both published and unpublished studies with epidemiological data on the prevalence of BSE among healthcare workers in Ethiopia.

After identifying key relevant primary studies, their references were also looked into. Similarly, other studies that cited them were viewed online. An Endnote software version 7x.2.1 was used to manage references.

### Inclusion criteria

#### Study setting

Only studies conducted in Ethiopia were included.

#### Design

Observational studies reporting breast self-examination practice and determinants of female healthcare workers were included.

#### Publication status

Both published and unpublished articles were considered.

#### Language

The articles published only in the English language were included.

#### Publication year

All publications reported up to April 24, 2020 were considered.

#### Exposure

Predictors/determinants of breast self-examination practice. The determinants are factors that increase or decrease the likelihood of breast self-examination practice.

#### Outcome

Female healthcare workers have ever performed breast self-examination for screening of breast cancer.

### Exclusion criteria

This review excluded articles and studies published in any language other than English. Review articles, case reports, case studies and simple descriptive studies without regression analyses were excluded. At the level of titles, titles that did not address breast self-examination practice among female healthcare workers were excluded. At the abstracts stage, studies that did not report factors associated with breast self-examination practice and qualitative studies were excluded. Full-text studies that did not report on the determinants of breast self-examination practice among female healthcare workers after multivariable regression analysis were excluded.

### Data extraction

Data were extracted from articles included in the review using a data extraction tool which was adapted from the Joanna Briggs Institute (JBI). Data were extracted after screening of titles, abstracts and the full texts of each primary studies included in this meta-analysis at least two times by the author. Any variance between the first and second data extraction was argued and resolved by extracting the data for the third times. Data were extracted for each article include: the name of first author, year of publication, study region and setting, study design, study participants, sample size, response rate, prevalence and risk factors with 95% confidence intervals.

### Risk of bias (quality) assessment

The quality of all articles selected in the review were assessed rigorously. To measure the risk of bias within the included studies, the methodological quality of each primary studies was assessed by using the Newcastle-Ottawa scale (NOS) tool adapted for cross-sectional studies quality assessments [43]. The author was assessed the quality of each primary studies thoroughly and repeatedly, at least two times. Any disagreements between the first and repeated levels of quality assessment was resolved through third times quality assessment. The assessment tool consists of 10 items (stars) in three main sections. The first section of the tool rated as five-star focuses on selection which is the methodological quality of each study (i.e., sample size, response rate, sampling technique and ascertainment of the exposure or risk factor). The second section of the tool focused on the comparability of the study (study controls for the most important factor and study control for any additional factor) with a possibility of two stars to be gained. The last section is concerned with assessment of the outcomes and statistical tests of the original study with a possibility of three stars to be gained. Finally, primary studies assessed with a score of ≥6 out of 10 were considered as achieving high quality, studies scored 5–6 out of 10 were considered as medium quality and studies scored ≤ 4 out of 10 were considered as low quality. The cut-off point for inclusion was declared after reviewing relevant literature.

### Outcome measures

The primary outcome variable of this review is breast self-examination practice which is measured by asking female HCWs wither they have ever performed breast self-examination for screening of cancer (yes/no). The second outcome of this study was to identify factors associated with breast self-examination practice among female healthcare workers which was measured in the form of the odds ratio (OR). Based on the binary outcome data reported by each study OR was calculated for each identified factor.

### Data synthesis and analysis

The extracted data were entered into a Microsoft Excel data base and then imported into STATA version 11 for further analysis. Tables and figures were used to summarize the selected studies. Meta-analysis was implemented for studies that provided the outcome and the determinants variables. Estimates of adjusted odds ratio with 95% confidence interval (CI) was considered as the measure of association for factors that determine breast self-examination practice of female healthcare workers. The pooled estimate of breast self-examination practice was estimated using a random effect model with 95% CI. Because of heterogeneity was exhibited among the included studies, random effect model was used during analysis. Heterogeneity between the studies was assessed with Cochran’s Q statistic and the I^2^ statistics. I^2^ values greater than 50% were considered as indicative of substantial heterogeneity [44]. Evidence of publication bias was assessed using the visual inspection of the asymmetry in funnel plots and Egger’s test [45]. Furthermore, subgroup analysis was done based on the profession that is Health Extension Workers (HEW) and Other HCW (Nurses, Midwives, Doctors, Pharmacies and others) to reduce the random variations among the point estimates of the primary study.

## Results

### Study selection

**Fig 1:**
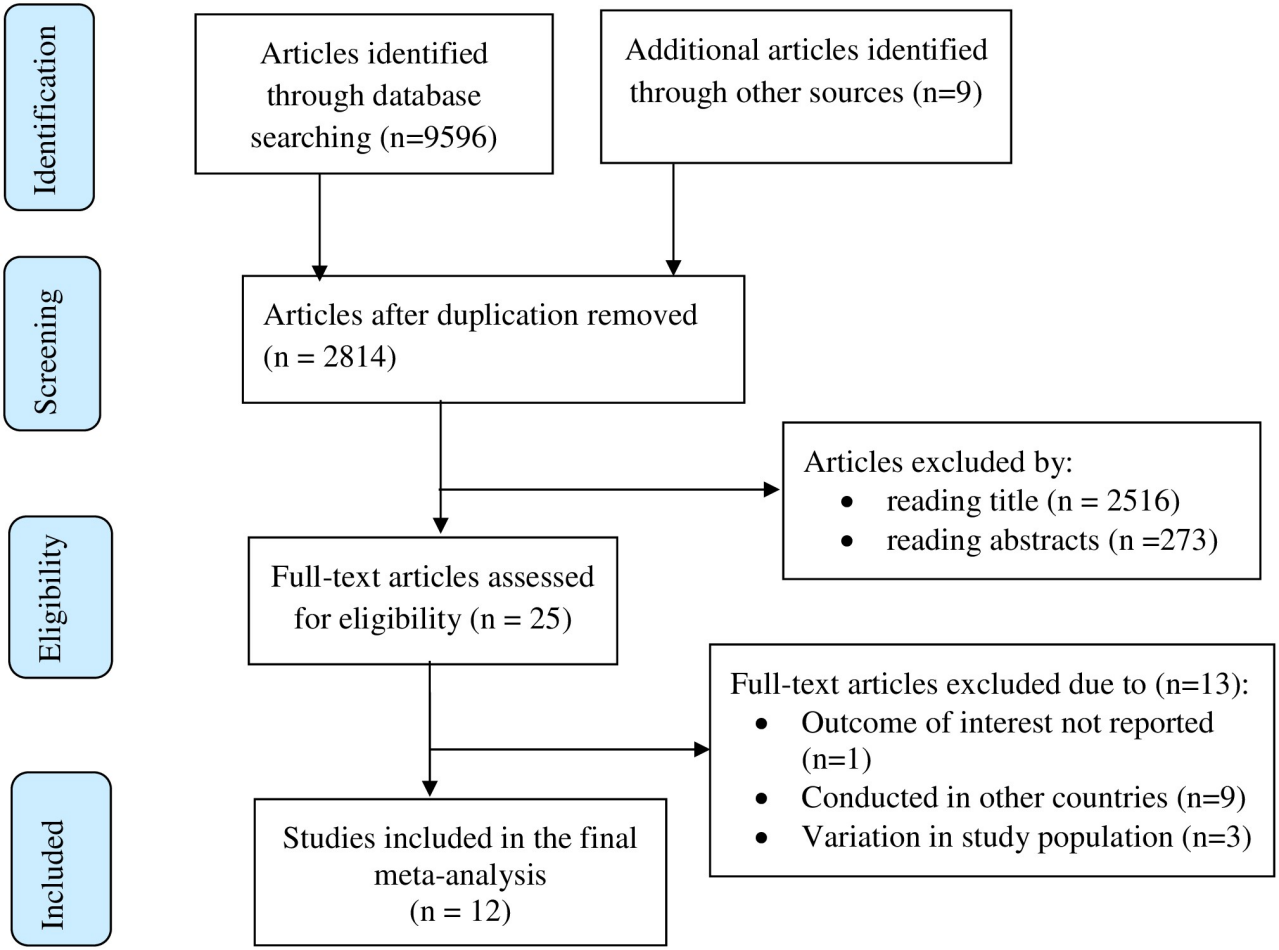
Flow chart of study selection for systematic review and meta-analysis of breast self-examination among female healthcare workers in Ethiopia, 2020.

The literature search strategy yielded 9,605 recorded articles. After removal of duplicates, 2,814 articles remained. After reading titles and abstracts, 2,789 articles were excluded. The studies were excluded because they did not address BSE practice among female healthcare workers, assessed BSE practice of women but not among female healthcare workers and conducted other than Ethiopia. Then, 25 full-text articles were assessed for eligibility. Of them 13 articles were excluded due to variation in study population, study locations and for not reporting the outcome of interest (S2 Table). Finally, 12 studies were included in the systematic review and meta-analysis (Fig 1).

### Study characteristics

All the included studies used cross-sectional study design. One [46] of the 12 studies were unpublished while the other 11 studies were published. In this systematic review and meta-analysis, a total of 4,129 participants were involved from 4,343 HCW, yielding a response rate of 95.1%. The sample size ranged from 167 in Gambella town [47] to 508 in Addis Ababa [48]. The response rate of the primary studies ranges from 89.2% to 100%. The highest prevalence of breast self-examination practice among HCWs (80.7%) was reported in western Ethiopia [49] while the lowest practice (32.5%) was reported from a study done in Debre tabor town [50]. Majority (n = 7) of the studies used self-administered questionnaires adapted from previous studies to assess breast self-examination practice and associated factors, and two studies do not explain where they adapt the questionnaires. Regarding geographical distribution of the studies, four studies were conducted in Addis Ababa, two studies in Southern Nations, Nationalities, and Peoples’ Region (SNNPR), two studies in Oromia, two studies in Amhara, one in Benishangul Gumuz region and one was done in Dire Dawa Administration. Regarding the study period, all the primary studies were conducted from 2012 to 2019 (Table 1).

**Table 1:** Descriptive summary of primary studies included in the systematic review and meta-analysis of breast self-examination practice and determinants among HCW in Ethiopia, 2020.

First author	Year	Region	Study area	Study design	Study population	Sample size	Response rate (%)	Prevalence (%)
Minasie A et al [51]	2017	SNNPR	Wolaita zone	Cross sectional	Health extension workers	281	100	45.6
Elias L et al [49]	2017	Oromia	Western Ethiopia	Cross sectional	Female health professionals	314	95.5	80.7
Muluken A et al [52]	2013	Amhara	West Gojam zone	Cross sectional	Health extension workers	403	98.0	37.2
Yosef Z et al [48]	2018	Addis Ababa	Addis Ababa	Cross sectional	Health extension workers	508	89.2	67.8
Seifadin A and Jibril D [53]	2019	Oromia	West Shoa zone	Cross sectional	Female healthcare workers	379	89.7	32.6
Wegene J [54]	2019	SNNPR	Hawassa University	Cross sectional	Female Nurses	196	91.8	71.1
Dagnechew D et al [47]	2019	Benishangul Gumuz	Gambella town	Cross sectional	Female healthcare workers	167	95.83	62.2
Imam D et al [55]	2019	Dire Dawa	Dire Dawa town	Cross sectional	Female healthcare professionals	387	97.0	38.1
Asrat H et al [50]	2019	Amhara	Debre Tabor Town	Cross sectional	Female healthcare workers	421	100	32.5
Seife T et al [56]	2012	Addis Ababa	Addis Ababa	Cross sectional	Female healthcare professionals	442	95.0	73.8
Teshome H et al [57]	2019	Addis Ababa	Addis Ababa	Cross sectional	Female healthcare professionals	422	92.2	77.9
Selamawit W et al [46]	2016	Addis Ababa	Addis Ababa	Cross sectional	Female health professionals	423	99.0	71.1

### Risk of bias (quality) assessment for the included studies

Risk of bias for each of the primary studies was conducted using the Newcastle-Ottawa scale tool adapted for cross-sectional studies as all included original studies were cross sectional. Of the total primary studies included in this systematic review and meta-analysis, the quality assessment summary showed that about-than three-fourth (n = 9, 75%) of the studies deemed high quality, and the reaming three (25%) of studies had medium quality. Furthermore, the quality assessment summary conducted for included studies indicated that there were no studies deemed to be low quality (Table 2).

**Table 2:** Quality assessment of primary studies included in the systematic review and meta-analysis of breast self-examination practice and determinants among HCW in Ethiopia, 2020.

Studies ID	Selection (Maximum of five star)	Comparability (Maximum two star)	Outcome assessment (Maximum of three stars)	Overall quality
Minasie A et al [51]	****	**	**	High
Elias L et al [49]	****	**	**	High
Muluken A et al [52]	*****	**	**	High
Yosef Z et al [48]	***	*	**	Medium
Seifadin A and Jibril D [53]	****	**	**	High
Wegene J [54]	***	*	**	Medium
Dagnechew D et al [47]	***	*	**	Medium
Imam D et al [55]	****	*	**	High
Asrat H et al [50]	****	*	**	High
Seife T et al [56]	****	**	***	High
Teshome H et al [57]	****	*	***	High
Selamawit W et al [46]	****	**	**	High

### Meta-analysis

**Fig 2:**
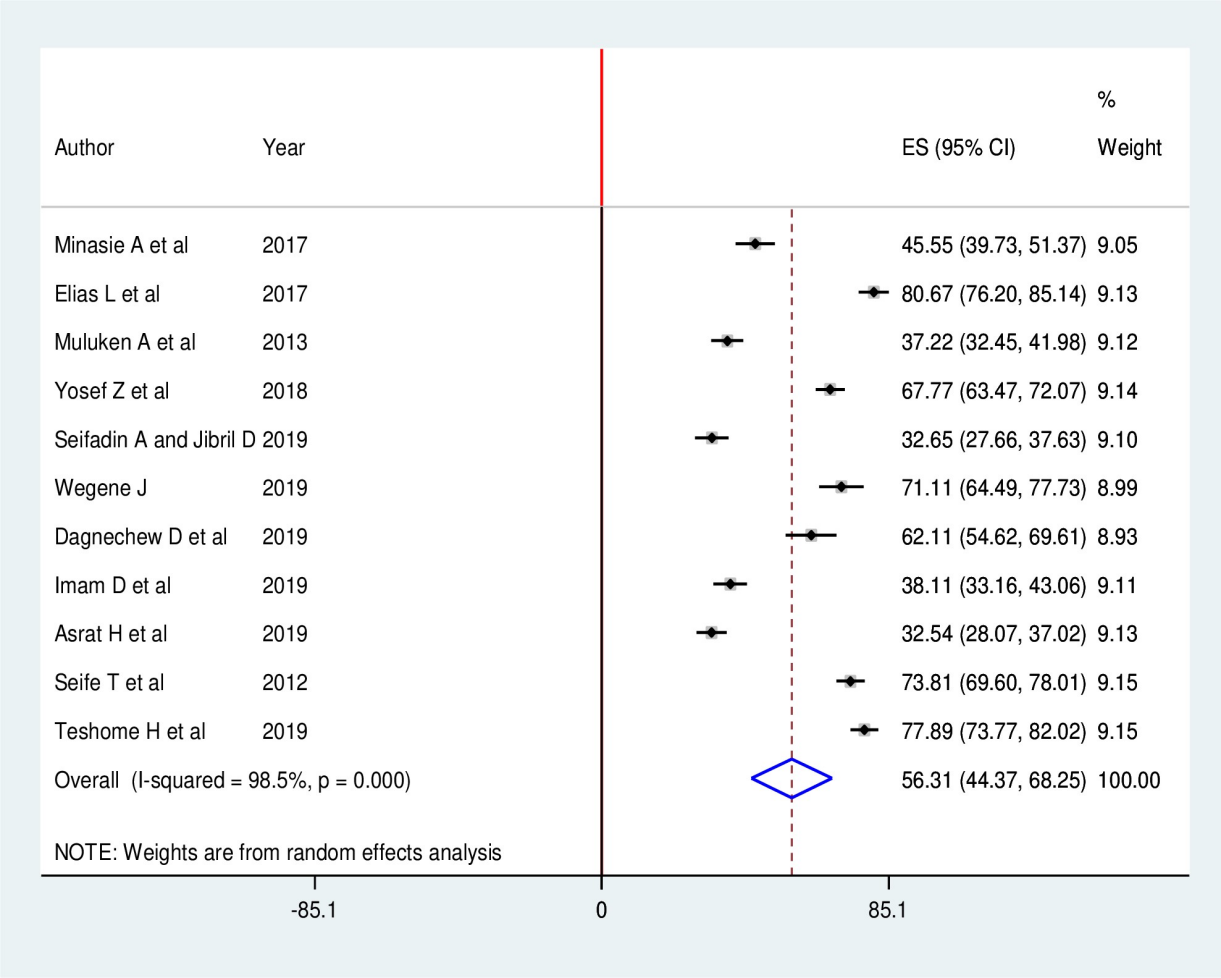
Forest plot of the pooled prevalence of breast self-examination practice among female healthcare workers in Ethiopia, 2020.

**Fig 3:**
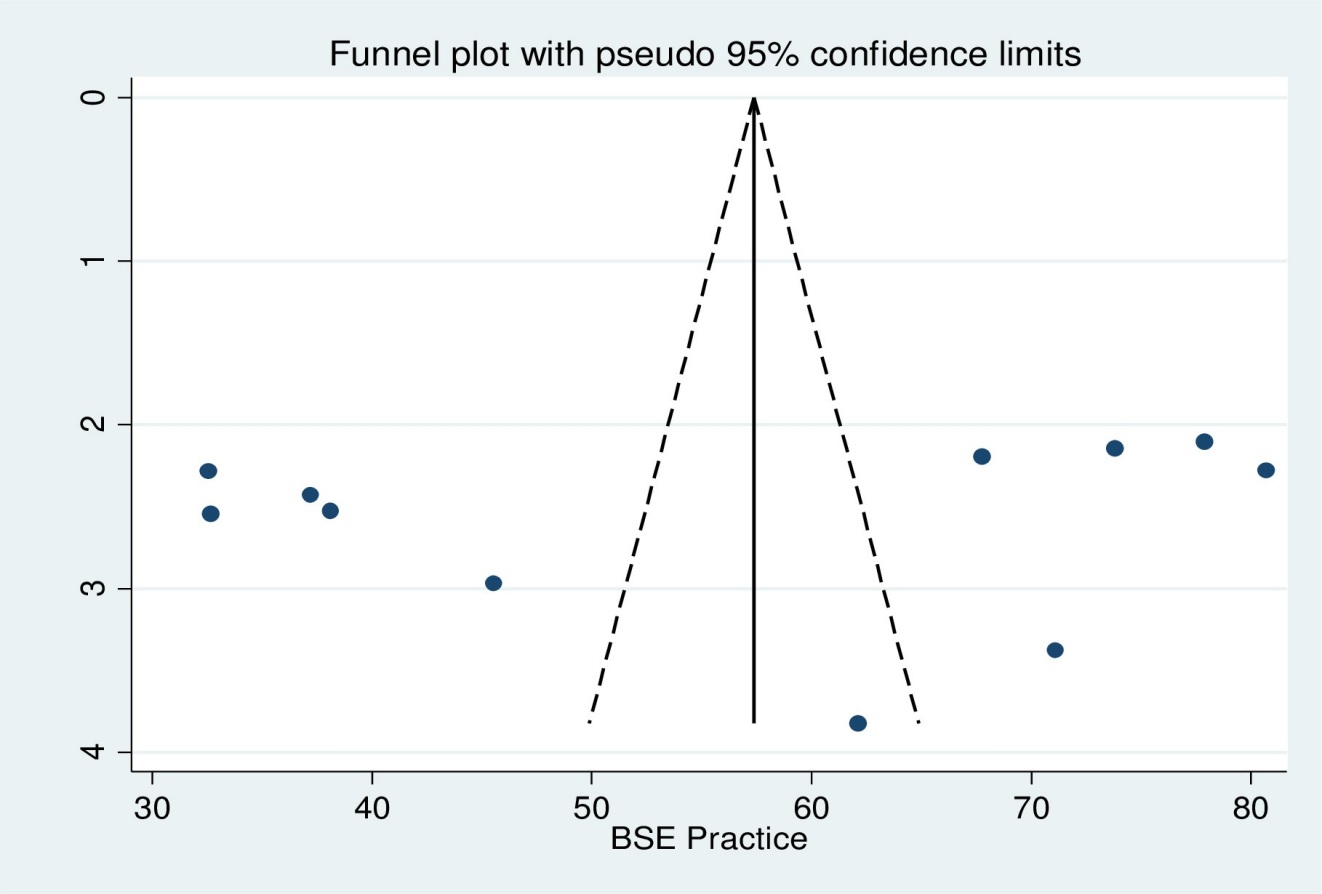
Graphic representation of publication bias using funnel plots of all included studies.

The pooled prevalence of breast self-examination practice among healthcare workers in Ethiopia was 56.31% (95% CI: 44.37, 68.25) There was extreme heterogeneity across the studies (I^2^ = 98.5, p < 0.001). Hence, random effect meta-analysis model was used to estimate the pooled prevalence of breast self-examination practice among healthcare workers in Ethiopia (Fig 2). Furthermore, subgroup analysis was done based on profession to reduce the random variations among the point estimates of the primary study. Publication bias among the included studies for this meta-analysis was checked using visual inspection of funnel plot and Egger’s test. Publication bias was not observed according to Egger’s test (P = 0.552) and the shape of funnel plots was symmetrical (Fig 3).

### Subgroup analysis

**Fig 4:**
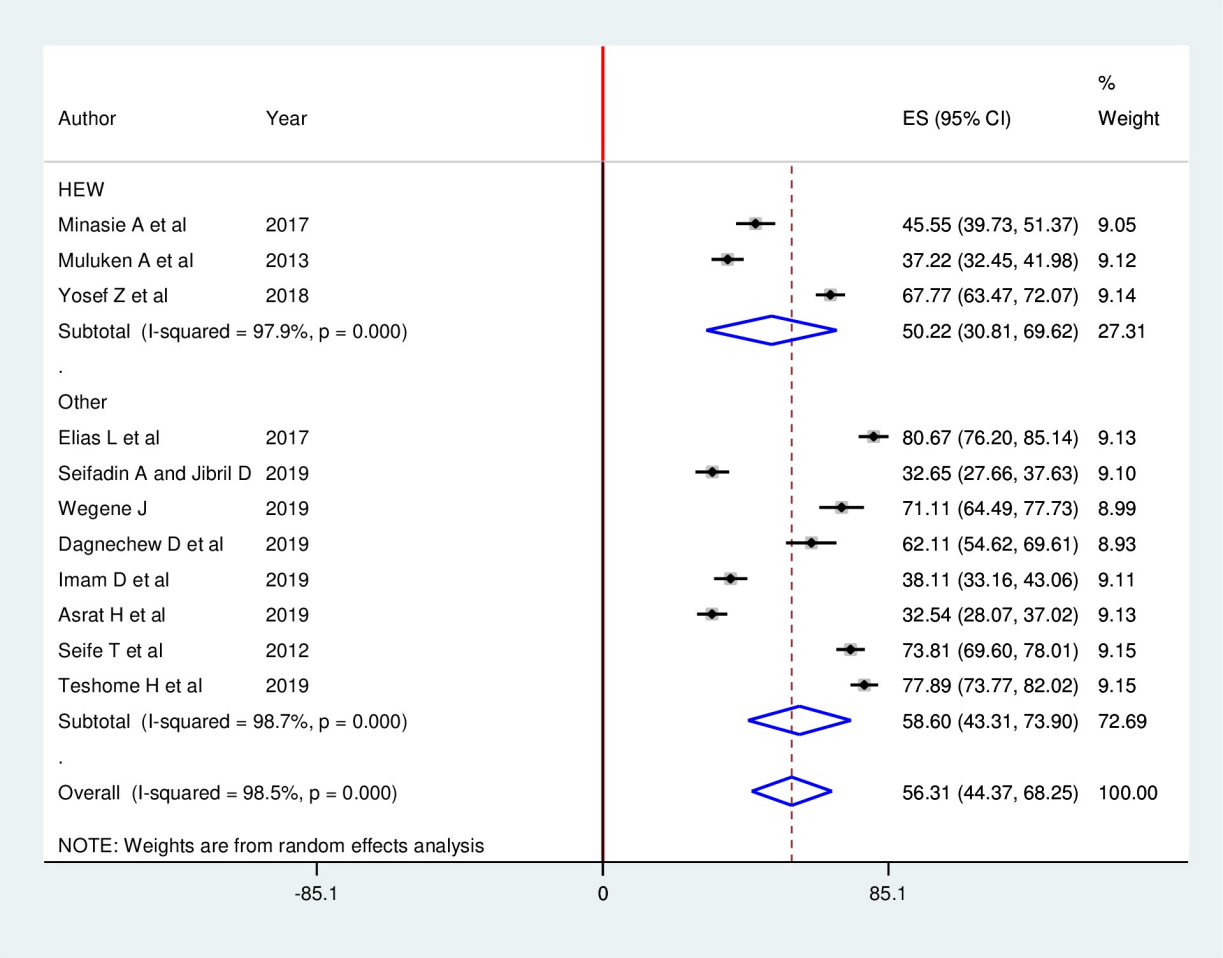
Sub-group meta-analysis by profession among female healthcare workers in Ethiopia.

In this meta-analysis, subgroup analysis was execute based on profession (HEW and Other HCWs). Accordingly, the result of this subgroup analysis found that studies conducted among other healthcare workers, 58.60% (95% CI: 43.31, 73.90) were slightly higher in breast self-examination practice as compared to those studies conducted among health extension workers, 50.22% (95% CI: 30.81, 69.62) (Fig 4).

### Factors associated with breast self-examination practice

In this review, some of the factors associated with breast self-examination practice were pooled quantitatively and some were not because of inconsistent grouping (classification) of the determinants with respect to the outcome (breast self-examination practice). Thus, those determinants reported in more than one primary studies were included in this meta-analysis.

**Fig 5:**
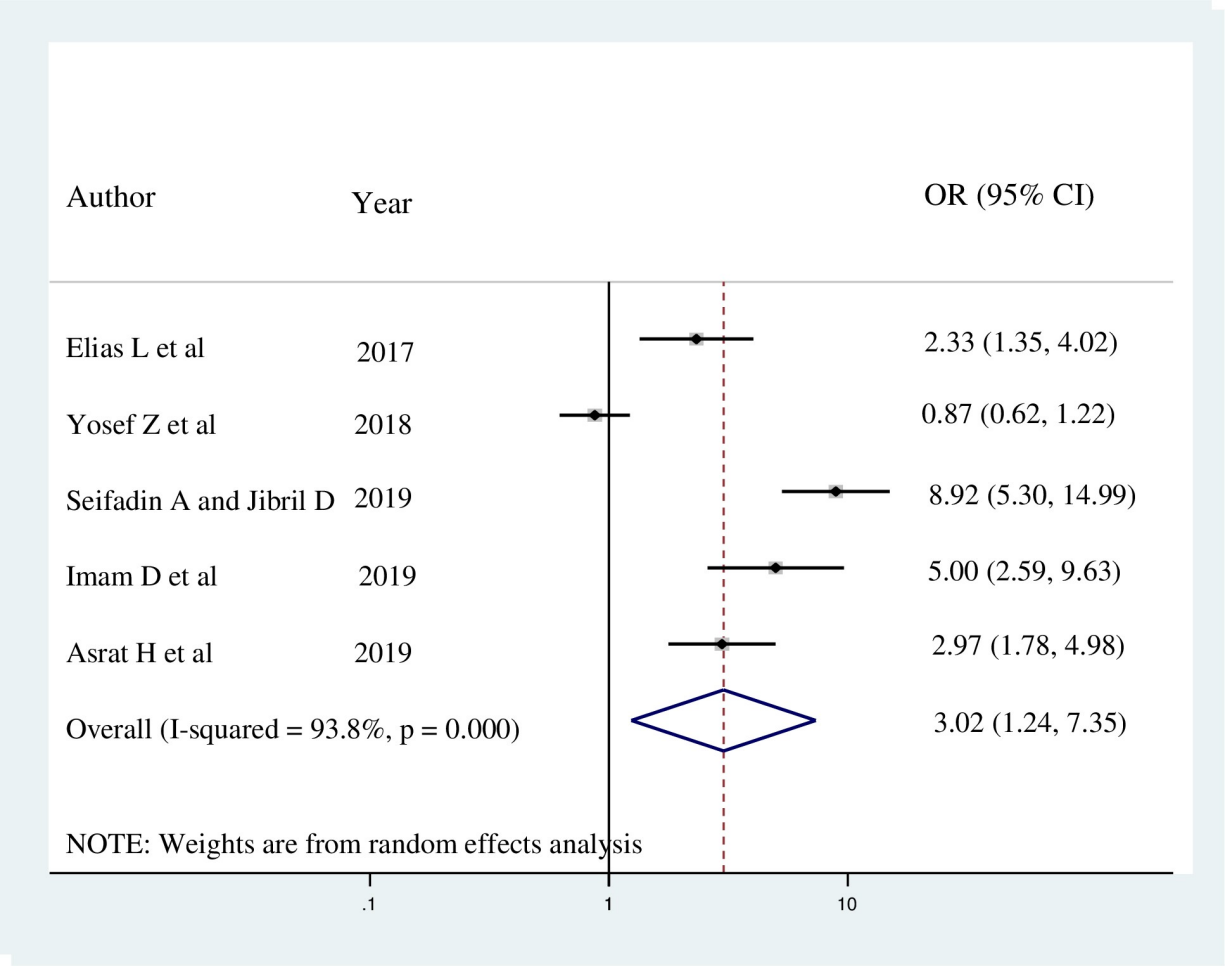
Forest plot showing the pooled odds ratio of the association between knowledge and breast self-examination practice among female healthcare workers in Ethiopia, 2020.

Five studies indicated that female healthcare workers who had good knowledge towards breast self-examination were more likely to practice BSE. The pooled odds ratio indicated that female healthcare workers who had good knowledge were 3.02 times more likely to practice breast self-examination than their counterparts (AOR = 3.02; 95% CI: 1.24, 7.35). Since high heterogeneity was exhibited (I^2^ = 93.8% and p < 0.001), a random effect meta-analysis model was used to determine the association (Fig 5).

**Fig 6:**
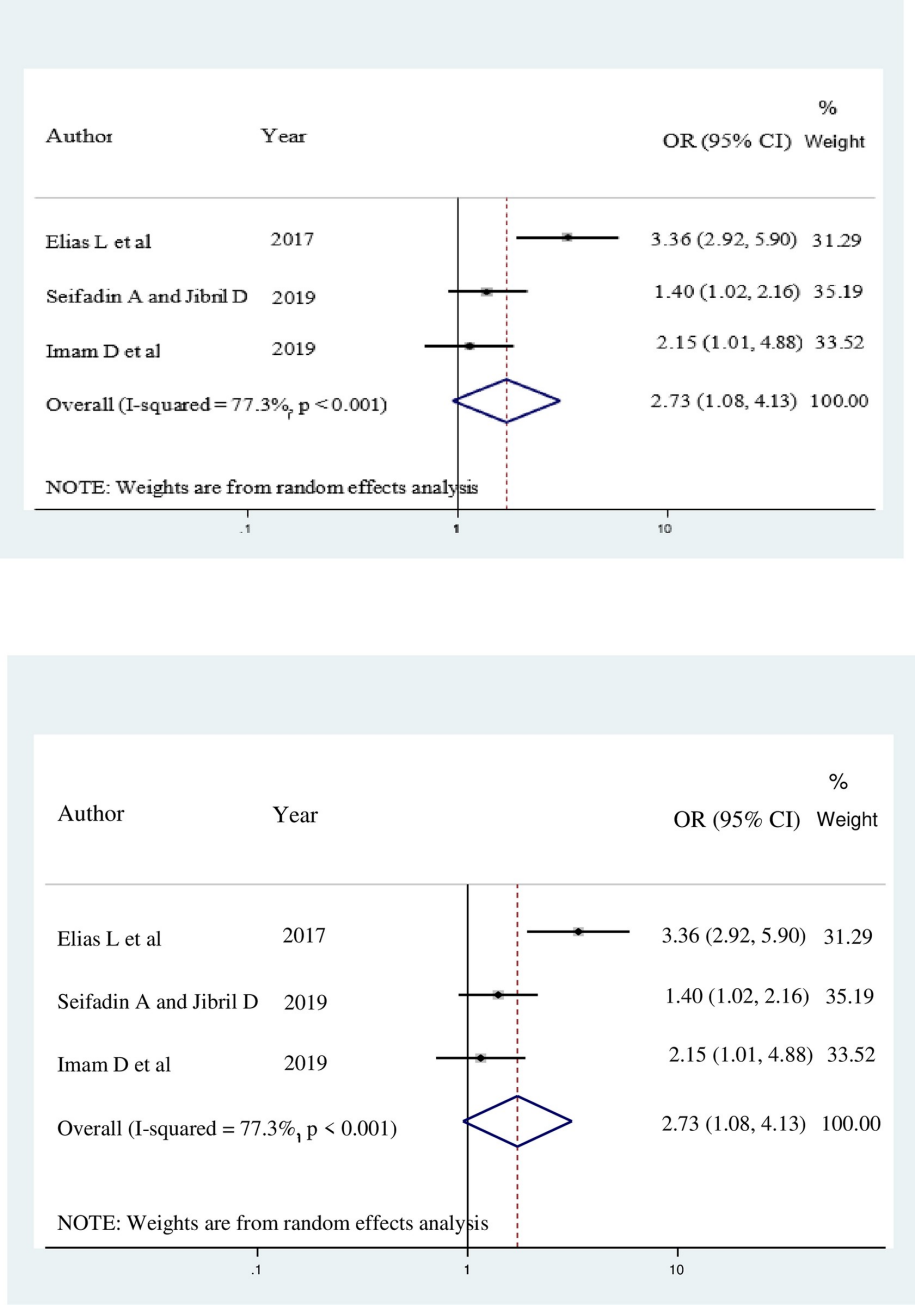
Forest plot showing the pooled odds ratio of the association between attitude and breast self-examination practice among female healthcare workers in Ethiopia, 2020.

Three studies also indicated that female healthcare workers’ attitude towards breast self-examination was strongly associated with BSE practice. The overall estimates revealed that female healthcare workers who had positive attitude (AOR = 2.73; 95% CI: 1.95, 4.13) were 2.73 times more likely to practice BSE as compared to their counterparts (Fig 6).

**Fig 7:**
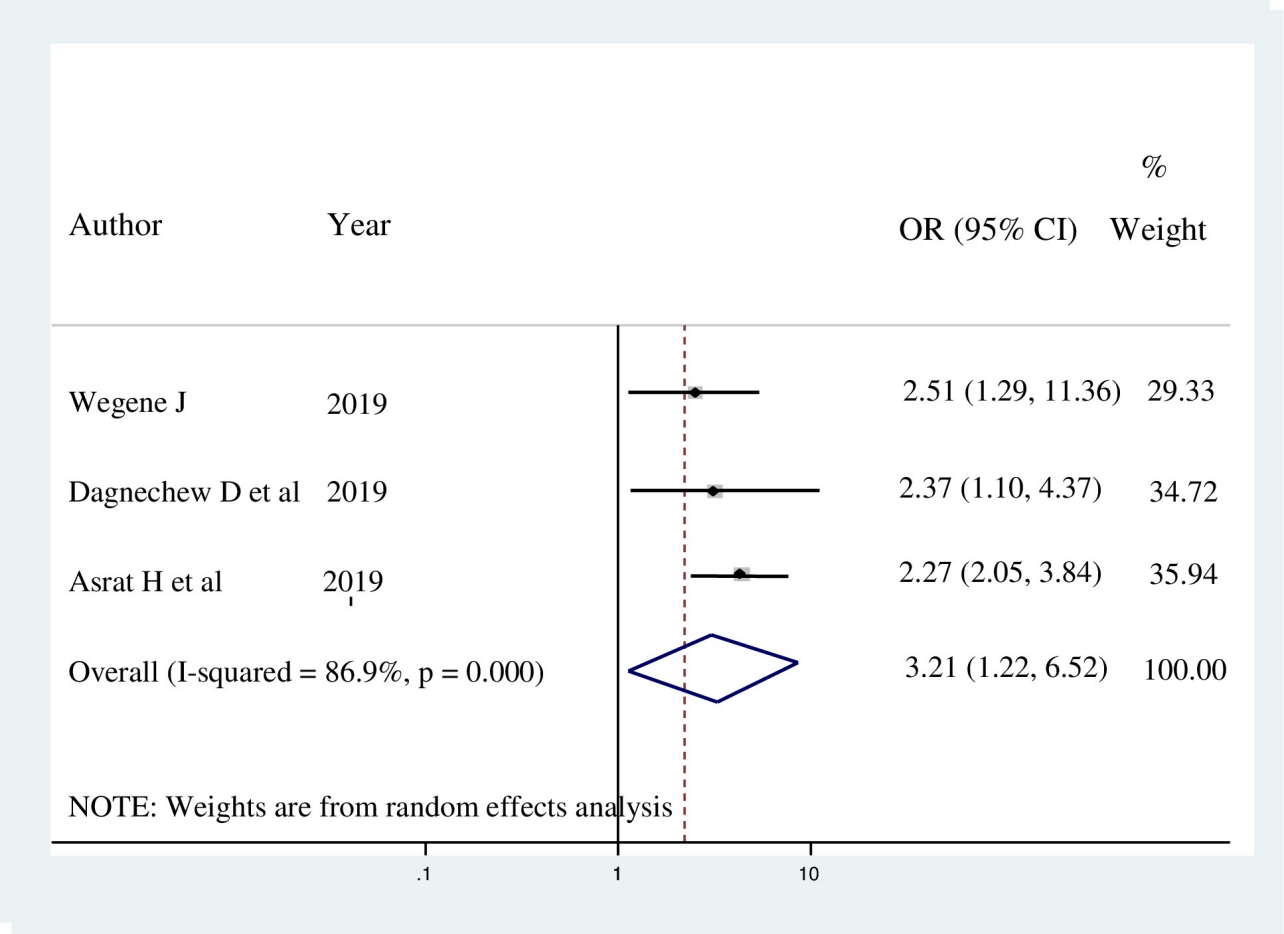
Forest plot showing the pooled odds ratio of the association between family history of breast cancer and breast self-examination practice among female healthcare workers in Ethiopia, 2020.

Family history of breast cancer was another significant factor associated with breast self-examination practice. Female healthcare workers who had family history of breast cancer were 3.21 times more likely to practice BSE compared to those who had no family history of breast cancer (AOR = 3.21; 95% CI: 1.22, 6.52) (Fig 7).

## Discussion

The rising burden of breast cancer will overwhelm the coping capacities of Ethiopia which is currently struggling with infectious diseases, child and maternal deaths [12, 58, 59]. To tackle breast cancer in time, healthcare workers should educate individuals to develop health protective and promoter behaviors. Teaching BSE at the individual level could improve awareness of breast cancer and lead to earlier stage at diagnosis. Thus, this meta-analysis was conducted to estimate the pooled prevalence of breast self-examination and to identify its determinants among female healthcare workers.

In this systematic review and meta-analysis, nearly half of female healthcare workers were not performed breast self-examination. This finding implies: the need of better enactment of breast cancer prevention programs; tendency of women to visit health facilities in late stage at diagnosis; and lower levels of breast cancer awareness and other cultural barriers to breast self-examination. Studies suggest that lower levels of breast cancer awareness could be improved through success of interventions targeted at raising awareness [29, 40, 60, 61]. The finding of this study also indicates the needs of evaluating existing breast cancer prevention programs by government workers and other stakeholders. This may be attributed to the role of BSE in breast cancer mortality reduction. Evidences showed that lower risk of mortality was found among women who reported practicing BSE before diagnosis [62, 63]. Furthermore, the finding suggests the need of training provision for health professionals regarding breast cancer and its screening methods to improve their knowledge, skill and motivation.

In the current meta-analysis, the pooled prevalence of breast self-examination practice among healthcare workers in Ethiopia was 56.31% (95% CI: 44.37, 68.25). Even if there was no comparable meta-analysis study conducted on this specific research question, the results of the finding is consistent with studies conducted in Egypt 56.4% [64] and in Singapore 63% [65]. However, this finding is lower than a study conducted in Nigeria 77.6% [66]. The possible reason could be attributed to the difference in study area; while this meta-analysis considered female healthcare workers working in both urban and rural areas, the previous study was focused on urban settings where better access to information is avail. Furthermore, the variation may be due to the differences in sociocultural values, norms, religious beliefs, and accessibility of information on breast cancer.

The subgroup analysis of this study indicated that the higher prevalence of breast self-examination practice was observed among other healthcare workers, 58.60% (95% CI: 43.31, 73.90) than HEW. The possible reason for this variation could be due to the differences in healthcare workers’ perceptions towards breast cancer and breast self-examination practice. Furthermore, the discrepancy could be due to the differences in access to mass media for further information on severity of the disease, and the level of skill on BSE in detecting any change at early stage.

This study identified that female healthcare workers who had good knowledge on BSE were about three times more likely to practice breast self-examination. This finding was in line with a meta-analyses conducted in Turkey [67] and other study carried out in Malaysia [68]. This might be explained by the fact that having knowledge could increase individuals’ self-confidence and gained experience; and this prompts female healthcare workers to practice breast self-examination. This highlights the need of training on breast cancer and its prevention programs for better implementation of breast self-examination. Literatures indicated that the individuals’ level of knowledge about health and diseases could allow them to appreciate the effects of health protective behaviors, and to develop positive health perceptions to reduce the barriers [67, 69].

Moreover, in this meta-analysis, participants’ attitude towards breast self-examination was significantly associated with BSE practice. Female healthcare workers who had positive attitude towards breast self-examination were about 2.73 times more likely to practice BSE. This finding is in line with a systematic review and meta-analysis conducted in Turkey [67]. This implies that the role of positive attitude towards breast self-examination in detecting breast cancer at an early stage is crucial. It also indispensable to promote health, exercise preventive interventions and BSE practice to succeed. It has been indicated that attitudes towards BSE can have significant impacts on early detection practices [29].

Family history of breast cancer was another significant factor associated with breast self-examination practice. Female healthcare workers who had family history of breast cancer were about 3.21 times more likely to practice BSE. This finding is in line with different studies [70–73]. This might be explained by the consciousness of individuals develop about the consequences of the disease may prompts them to practice BSE. Additionally, it could be due to fear of acquire a life-threatening disease from a family make them cautious. Moreover, individuals with family history of breast cancer see themselves under risk for the disease and believe in the importance of screening for early diagnosis [74].

### Limitations of the study

This systematic review and meta-analysis was considered only English-language articles; this could result in language bias. In addition, the heterogeneity across selected studies was high, which further reflects the need to address important sources of heterogeneity. Subsequently, the review process of this study was conducted with a single author which may raise the question about the methodological rigor of the review. Regarding sample size, some of the studies included in this review had not as such large sample size and this may influence the estimated report. Since, all the included studies were cross-sectional in nature; the outcome variables might be affected by other confounding variables. Furthermore, this meta-analysis represented only studies reported from four regions and two administrative town of the country, which could affect the estimated prevalence reported.

## Conclusion

This meta-analysis found that the prevalence of breast self-examination was relatively low, with slightly more than 1 in 2 female healthcare workers perform breast self-examination practice. Female healthcare worker’s knowledge, attitude towards BSE and family history of breast cancer were identified factors significantly associated with breast self-examination practice among female healthcare workers. The finding of this study suggests the need of deployed and strengthening of early diagnosis of breast cancer and control strategies with a collaborative work of policymakers, programmers and other concerned stakeholders. It also suggests that raising breast cancer and breast self-examination awareness through community awareness programs should be provided to promote the level of knowledge. Furthermore, public health disease preventive behaviors (breast self-examination) should be considered as an important and feasible preventive strategies of breast cancer.

## Supporting information

S1 Table Preferred Reporting Items for Systematic Reviews and Meta-Analyses (PRISMA) checklist. (DOC)

S2 Table List of excluded studies references and reasons for exclusion. (DOCX)

## Abbreviations

AOR: Adjusted odd ratio
BSE: breast self-examination
CI: Confidence intervals
HCWs: healthcare workers
OR: Odds ratio
SSA: Sab Saharan Africa
