# Supplementary material for: Breast self-examination practice and associated factors among female healthcare workers in Ethiopia: A systematic review and meta-analysis
Source: PLoS One. 2020 Nov 10;15(11):e0241961. doi: 10.1371/journal.pone.0241961 (PMC7654829; doi:10.1371/journal.pone.0241961)
Supplement: S2 Table — (DOCX) [file pone.0241961.s002.docx]

**S2 Table. List of excluded studies references and reasons for exclusion**

| **S.No** | **Study** | **Reason for exclusion** |
| --- | --- | --- |
| 1 | Mitra Savabi-Esfahani et al. Role Playing for Improving Women’s Knowledge of Breast Cancer Screening and Performance of Breast Self-Examination Asian Pacific Journal of Cancer Prevention, Vol 18. DOI:10.22034/APJCP.2017.18.9.2501 | Conducted in other countries |
| 2 | Semarya Berhe Lemlem, Worknish Sinishaw, Mignote Hailu, Mesfin Abebe, and Alemseged Aregay. Assessment of Knowledge of Breast Cancer and Screening Methods among Nurses in University Hospitals. Addis Ababa, Ethiopia. 2011. | The outcome of interests was not reported |
| 3 | Birhane et al. Predictors of breast self – examination among female teachers in Ethiopia using health belief model. Archives of Public Health (2015) 73:39 | Variation in study population  (Focused only on school teachers) |
| 4 | Akhtari Z, Mehrnoosh, Juni, Muhamad H, Abd Manaf, et al. Mehrnoosh A. and et al. Knowledge on breast cancer and practice of breast self-examination among selected female. Medical and Health Science Journal, MHSJ. 2011; 7:49–56. | Conducted in other countries |
| 5 | Befikadu Legesse TG. Knowledge on breast cancer and its prevention among women household heads in Northern Ethiopia, vol. 4; 2014. p. 9. | Variation in study population (Focused on general population) |
| 6 | Zografos GC, Sergentanis TN, Zagouri F, Papadimitriou CA, Domeyer P, Kontogianni PN, et al. Breast self-examination and adherence to mammographic follow-up: an intriguing diptych after benign breast biopsy. Eur J Cancer Prev. 2010; 19: 71–2. | Conducted in other countries |
| 7 | Firealem Solomon, Emabet Berhane and Kalkidan Wondwossen. Practice of Breast Self-Examination and Associated Factors Among Women in Dire Dawa Town, Ethiopia, 2018. | Variation in study population (Focused on general population) |
| 8 | Avci IA. Factors associated with breast self-examination practices and beliefs in female workers at a Muslim community. Eur J Oncol Nurs. 2008;12(2):127–33. | Conducted in other countries |
| 10 | Demirkiran F, Balkaya NA, Memis S, Turk G, Ozvurmaz S, Tuncyurek P. How do nurses and teachers perform breast self-examination: are they reliable sources of information? BMC Public Health. 2007;7(1):96. | Conducted in other countries |
| 11 | Care C. Correlates of Breast Self-Examination: application of the Trans theoretical model of change and the health belief model. 2005;1–105. | Conducted in other countries |
| 12 | Kaushalendra Mani Tripathi., et al. “Assessment of the Knowledge of Breast Cancer and Breast Self- Examination”. EC  Paediatrics 7.12 (2018): 1212-1229. | Conducted in other countries |
| 13 | Bashirian S, Barati M, Mohammadi Y, Moaddabshoar L, Dogonchi M. An Application of the Protection Motivation Theory to  Predict Breast Self-Examination Behavior among Female Healthcare Workers. Eur J Breast Health 2019; 15(2): 90-97. | Conducted in other countries |
